# A randomized, sham-controlled trial of high-definition transcranial direct current stimulation on the right orbital frontal cortex in children and adolescents with attention-deficit hyperactivity disorder

**DOI:** 10.3389/fpsyt.2023.987093

**Published:** 2023-02-13

**Authors:** Yi-chao Wang, Jun Liu, Yan-chun Wu, Yan Wei, Hong-jing Xie, Tao Zhang, Zhen Zhang

**Affiliations:** ^1^Affiliated Mental Health Center & Hangzhou Seventh People's Hospital, Zhejiang University School of Medicine, Hangzhou, Zhejiang, China; ^2^Zhenjiang Mental Health Center (The Fifth People's Hospital of Zhenjiang City), Zhenjiang, Jiangsu, China

**Keywords:** attention-deficit hyperactivity disorder (ADHD), orbital frontal cortex (OFC), high-definition transcranial direct current stimulation (HD-tDCS), executive function (EF), IVA-CPT

## Abstract

**Objective:**

This study aimed to find out the clinical and cognitive effects of high-definition transcranial direct current stimulation (HD-tDCS) on the right orbital frontal cortex (OFC) in the treatment of attention deficit hyperactivity disorder (ADHD).

**Methods:**

A total of 56 patients with ADHD were recruited as subjects and completely and randomly divided into the HD-tDCS group and the Sham group. A 1.0 mA anode current was applied to the right OFC. The HD-tDCS group received real stimulation, while the Sham group received sham stimulation in 10 sessions of treatment. ADHD symptom assessment (the SNAP-IV Rating Scale and the Perceived Stress Questionnaire) was carried out before treatment, after the 5th and 10th stimuli, and at the 6th week after the end of all stimulations, while the cognitive effect was assessed by the Integrated Visual and Auditory Continuous Performance Test (IVA-CPT), the Stroop Color and Word Test (Stroop), and the Tower of Hanoi (TOH). Repeated-measure ANOVA was used to find out the results of both groups before and after treatment.

**Results:**

A total of 47 patients completed all sessions and evaluations. Their SNAP-IV score, their PSQ score, the mean visual and auditory reaction times by IVA-CPT, the interference RT of Stroop Color and Word, and the number of completed steps of TOH did not change with intervention time before and after treatment (*P* > 0.0031). However, the integrated visual and audiovisual commission errors and the TOH completion time results of the HD-tDCS group were significantly decreased after the 5th intervention, the 10th intervention, and the 6th week of intervention follow-up compared to the Sham group (*P* < 0.0031).

**Conclusion:**

This study draws cautious conclusions that HD-tDCS does not significantly alleviate the overall symptoms of patients with ADHD but leads to significant improvements in the cognitive measures of attention maintenance. The study also attempted to fill in the gaps in research studies on HD-tDCS stimulation of the right OFC.

**Clinical trial registration:**

ChiCTR2200062616.

## Introduction

Attention deficit hyperactivity disorder (ADHD) is a common neurodevelopmental disease among children and adolescents. Its core defects are mainly characterized by attention disorder, hyperactivity, impulsivity, and other clinical symptoms. Many children and adolescents with ADHD are associated with learning difficulties (1), with some having marked difficulties with emotional control (2). Neuropsychologists have found that there are performance defects in psychological processes with ADHD (3). Although ADHD is a complex and heterogeneous disorder (4), children and adolescents with ADHD often show performance impairments on tasks that measure some form of executive processes (5). ADHD is associated with deficits across a range of cognitive domains, such as arousal, executive functions, behavioral inhibition, motivation, set-shifting, and working memory (6). A recent meta-meta-analysis involved 34 meta-analyses of neurocognitive ADHD profiles (all ages) concerning 12 neurocognitive domains. Patients with ADHD have moderate impairments in multiple domains, including working memory, reaction time variability, inhibitory control, cognitive flexibility, intelligence/achievement, and planning/organization (7).

Transcranial direct current stimulation (tDCS) is a non-invasive brain stimulation therapy. Weak current (0.5–2.0 mA) affects specific brain regions through the scalp, which acts as electrodes. Anodal transcranial direct current stimulation (ATDCS) refers to electrical current flow from the anodic electrode to the target brain region, which increases the target brain region excitation. Cathodal transcranial direct current stimulation (CTDCS) refers to electrical current flowing from the cathode electrode to the target brain region, which decreases the target brain region excitation (8). This excitability change is caused by the change in the resting membrane potential in the relevant region (9). There was a prolonged effect after 30 min of the tDCS stimulation (10), and the effect even lasted several months after repeated tDCS stimulation (11). From fMRI observation, tDCS stimulation of the prefrontal lobe improved network connectivity at rest (12). The weak current of tDCS regulates cortical excitability and spontaneous neural activity by stimulating the corresponding cerebral cortex region, thus improving the functional abnormalities of the corresponding brain regions and showing good safety and tolerability (13).

Among the existing tDCS research, there are many studies on the dorsolateral prefrontal cortex (DLPFC), some of which have achieved positive results. Allenby believes that ATDCS can reduce the reaction time of Stop-Signal Tasks, which leads to the conclusion that tDCS can alleviate subject impulsivity and delayed gratification difficulties (14). Nejati suggested that the stimulation of the right DLPFC with ATDCS could improve persistent inhibition and partial interference control (15). A combined stimulation of the DLPFC and the orbitofrontal cortex (OFC) can reduce reaction time, improve cognitive flexibility, and improve working memory (16). According to recent research, Leffa et al. performed daily sessions of 30 min of home-based tDCS for 4 weeks on 64 participants with ADHD, totaling 28 sessions, with 2.0 mA anodal-right and cathodal-left prefrontal stimulations with 35-cm^2^ carbon electrodes. The efficacy results assessed by the inattentive scores of the clinician-administered versions of the Adult ADHD Self-report Scale (CASRS-I) show decreased symptoms of inattention in the active tDCS group over the three assessments compared to the sham tDCS group (17). However, according to several research studies, there is no evidence that tDCS can improve the response inhibition ability (18) and the sustained attention (19) of patients with ADHD. Some scholars suggested that the clinical efficacy and cognitive effects of tDCS in the treatment of ADHD, whether the inferior frontal gyrus (IFG) or the DLPFC, still need to be further verified in future studies (20, 21).

Traditional tDCS stimulation results in the diffusion and distribution of current in a wide range of brain regions, which may not be able to display the maximum current density directly below the electrode, leading to inaccurate positioning of tDCS stimulation. In recent years, the new high-definition tDCS (HD-tDCS) has been proposed to solve the problem of traditional tDCS in affecting the stimulation target to the extra brain regions. The HD-tDCS stimulation current is limited to the region below the electrode and thus improves the accuracy. This ensures high-current density in the main target region, minimizes the stimulation of the non-target regions, and reduces the risk of side effects. This shows that the same effect can be achieved by stimulating the corresponding brain regions with less current than conventional tDCS. Researchers stimulated the right IFG of 15 subjects with ADHD aged 10–16 years with HD-tDCS of 0.5 mA and evaluated the stimulation by the N-back test and event-related potential P300/N200. The results showed that, compared with the traditional tDCS stimulation of 1.0 mA, HD-tDCS also improved working memory and inhibitory control (22). Some researchers believe that HD-tDCS should be set as a further topic to study (23).

Orbito-frontal cortico-striato-thalamo-cortical (OFCSTC) loops, also known as the impulse/force loop, are applied in the control of impulsive behavior (24). The nerve fibers of the loop originate from the OFC and extend into the inferior caudate nucleus, then travel to the thalamus, and finally return to the OFC. The inactivation of this circuit leads to impulse control difficulties and emotional processing disorders. The OFC dysfunction was significantly associated with the severity of impulsive (25) and obsessive behavior (24). Impulsive symptoms of ADHD include excessive speech, unthinking interruptions, blurting out words, and unwillingness to wait in order, all of which involve this loop. The cortical thickness of the OFC in patients with ADHD was significantly lower than that in healthy controls (26). A Structural Covariance Network (SCN) analysis shows that the volume of gray matter on the right side of the OFC of patients with ADHD decreased significantly (27). In addition, structurally, a meta-analysis of whole-brain voxel-based morphometry (VBM) showed disorder-specific gray matter volume (GMV) abnormality in the OFC in ADHD (28). The fMRI scans showed that right OFC activation significantly decreased in patients with high-risk behavioral tendencies in the Go/NO-Go tasks (29). In addition, fMRI showed that the activation of the right OFC was associated with emotion-based risk tasks in negative emergencies, reflecting that risk-taking was associated with the ability of emotion-based risk control (30). Boys with ADHD showed disorder-specific underactivation in the OFC (31). High-resolution fMRI showed that adolescent patients with ADHD display enhanced OFC signaling of future rewards and that these increased reward-related responses were correlated with the severity of hyperactivity/impulsivity (32). Decreased cognitive capacity related to hyperactivity and impulsivity was associated with reduced OFC activity during reward expectation in patients with ADHD (28).

Although OFC was not a sufficiently activated region to be underactivated in recent fMRI meta-analyses of ADHD (33–35), there was evidence from the aforementioned studies for OFC underactivation, mainly in terms of rewards or emotions. Furthermore, impulse control difficulties and emotion-processing disorders based on OFCSTC could affect cognitive performance. In other words, relatively higher cognitive ability was associated with normalized OFC responses (32). Therefore, in this study, we hypothesized that HD-tDCS of the right OFC could alleviate the clinical symptoms, impulse control difficulties, and emotion processing of patients with ADHD to further improve their performance on cognitive tasks such as maintaining attention and inhibitory control and then test this hypothesis using a randomized, sham-controlled study.

## Materials and methods

### Research subjects

#### Inclusion criteria

Subjects included patients with ADHD who visited the general outpatient department and the children's outpatient department of the Zhenjiang Mental Health Center between March 2020 and November 2021. The patients were children and adolescents aged 8–18 years. They were diagnosed and reviewed by a senior associate chief physician or a chief physician of the department of psychiatry. All subjects met the diagnostic criteria of ADHD of the validated screening and diagnostic instrument: the Diagnostic and Statistical Manual of Mental Disorders (DSM-5) and The International Classification of Diseases (ICD-10). Simultaneously, the subtypes of ADHD were also classified, such as inattentive, hyperactive-impulsive, or the combined subtype; The Wechsler Intelligence Scale for Children-IV Chinese version was administered to every subject, with a total IQ ≥ 80 (36). All children were of Chinese Han origin and were right-handed.

#### Exclusion criteria

Contraindications for tDCS treatment include patients with metal device implants (such as the cochlear implant, the artery clamp, and the pacemaker); history of brain trauma or cerebrovascular accident, intracranial hypertension, skull defects, epilepsy, and other serious neurological, circulatory, endocrine, and other physical diseases; audio-visual impairments and color blindness, color weakness, or narrow-angle glaucoma. The abovementioned contraindications were excluded by inquiring and collecting medical history, conducting an electrocardiogram (ECG), electroencephalogram (EEG), cranial CT, and blood routine and biochemical examinations. All subjects were evaluated for no comorbidities of other mental disorders with validated screening and diagnostic instruments: DSM-5 and ICD-10, such as substance abuse/dependence, conduct disorders, personality disorders, autism, Tourette's disorder, and obsessive-compulsive disorder. Patients who had used any medication (methylphenidate, atomoxetine, etc.) in the past and recently to treat ADHD or who received other brain stimulation (transcranial magnetic stimulation, electroconvulsive shock, etc.) were also excluded.

**Figure 1:**
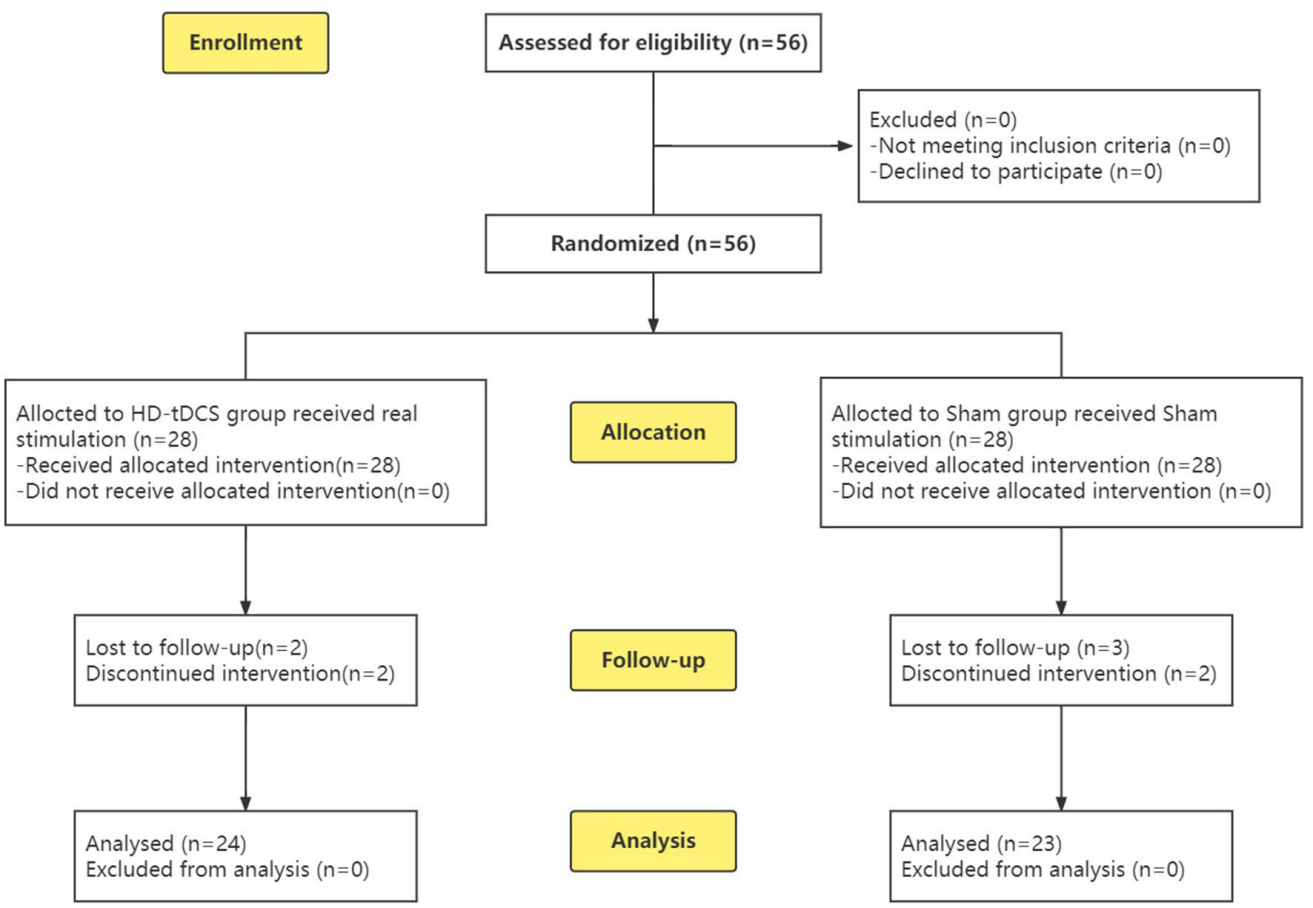
CONSORT flow diagram (38) of this RCT from enrolment, intervention allocation, follow-up and analysis.

To calculate the sample size, we used G^*^Power (37) with the following settings: effect size *f* = 0.25, α level = 0.05, power = 0.8, and correlation among repeated measures = 0.5. The minimum sample size was found to be *n* = 44. To prevent a potentially large number of dropouts, a total of 56 subjects were recruited, including 33 boys and 23 girls. A completely randomized experimental design was adopted, and the subjects were divided into two groups according to age through a random number table: the HD-tDCS group and the Sham group (Figure 1). A general information questionnaire was developed, including age, gender, educational years, whether the subject comes from a single-parent family, age of onset, and disease. Both the participants and their guardians were informed of this study, and signed informed consent was obtained. The study was approved by the Ethics Committee of Zhenjiang Mental Health Center. This trial was conducted in accordance with the Declaration of Helsinki and the Consolidated Standards of Reporting Trials (CONSORT) guidelines (38).

### Methods

While the HD-tDCS group received real stimulation, the Sham group received sham stimulation. Before receiving stimulation (T0), all subjects underwent ADHD symptom assessments (SNAP-IV rating scale, Conners Parents Questionnaire) and cognitive task [Integrated Visual and Auditory Continuous Performance Test (IVA-CPT), Stroop, Tower of Hanoi (TOH)] assessments to collect baseline data. Then, the subjects underwent either HD-tDCS stimulation or sham stimulation. The above ADHD symptom and cognitive task assessments were repeated for all subjects after the 5th stimulus (T1), the 10th stimulus (T2), and at the 6th-week follow-up after the end of all stimuli (T3).

### HD-tDCS

HD-tDCS uses a multichannel stimulator (Soterix Medical, 4 × 1-C3A, USA) that uses a constant direct current stimulator of conventional tDCS (Soterix Medical, 1 × 1 Low-Intensity Transcranial DC Stimulator, 1300A, USA), delivers, and converts it to high-definition stimulation. Conventional tDCS produces diffuse brain currents. The electrodes of HD-tDCS are arranged in a 4 × 1 ring on the skull, producing a more concentrated and precise current that is confined to the return electrode ring.

#### Stimulation site

**Figure 2:**
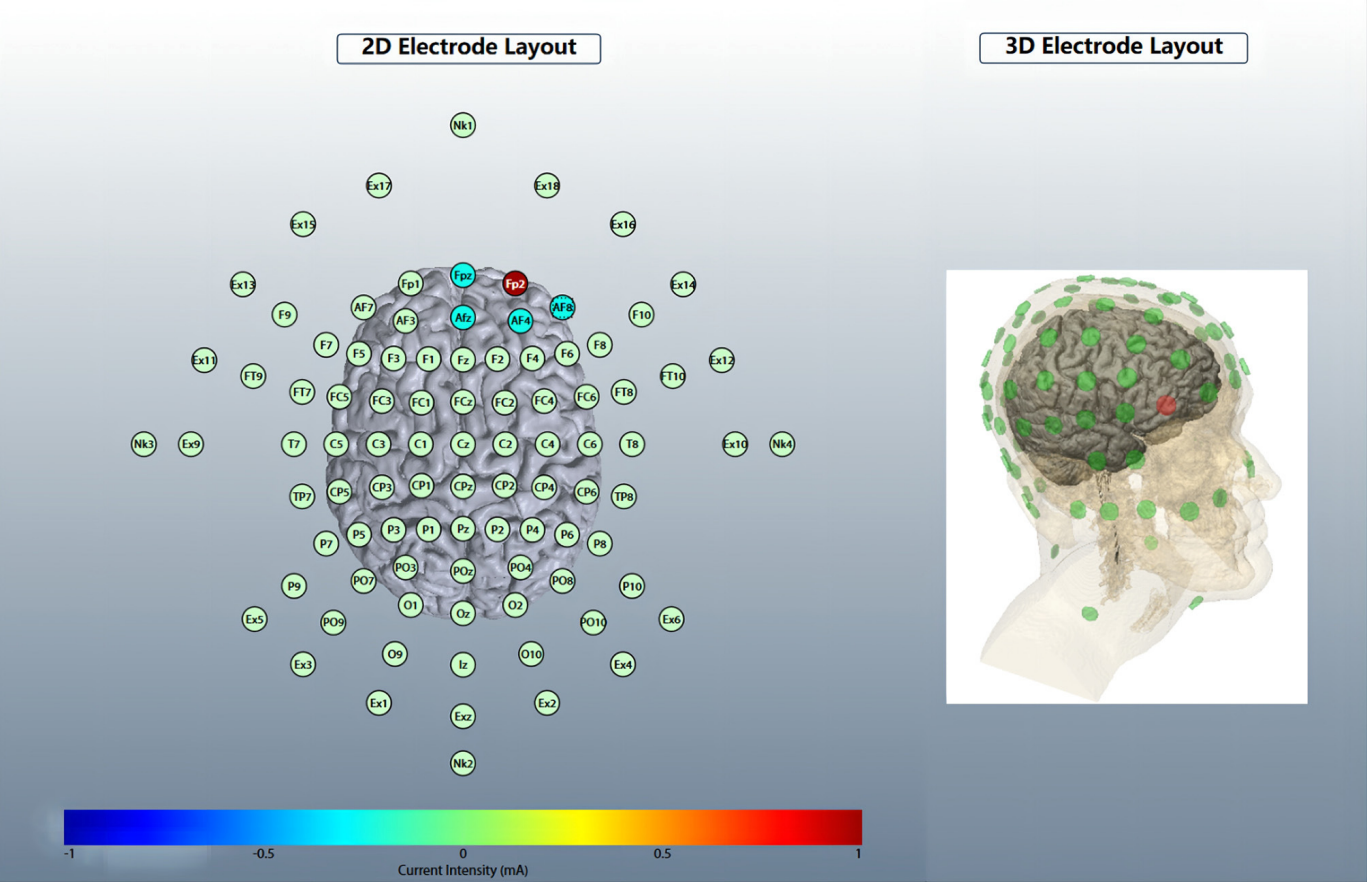
HD-tDCS electrode layout. An anodic electrode was placed at the center: right orbitofrontal cortex (OFC), corresponding to Fp2; four cathode electrodes are placed square around the anode, corresponding to Fpz, Afz, AF4, and AF8.

**Figure 3:**
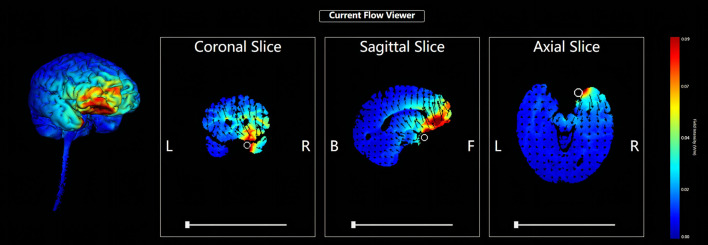
HD-tDCS electric field simulation. The intensity of the simulated field is indicated by the color bar, the arrow points to the direction of the current, and the length represents the current intensity.

Five circular Ag/AgCl electrodes with a diameter of 1 cm were placed, and one anode electrode was placed on the center: the right OFC, corresponding to the standard electrode location of the International Electroencephalogram Society 10/20 System: Fp2; four cathode electrodes (i.e., return electrodes) are placed in a square around the anode, about 5 cm away from the anode, and corresponding to Fpz, Afz, AF4, and AF8 (Figure 2). Electric field simulation was performed using the HD-Explorer software (Soterix Medical, USA). The intensity of the simulated field is indicated by the color bar, the arrow points to the direction of the current, and the length represents the current intensity (Figure 3).

#### Stimulation parameters

The HD-tDCS anode current intensity was 1.0 mA, and each stimulation lasted for 20 min during which there was 30 s of current increase time and 30 s of current decrease time, one time a day for five consecutive days, 2 days of rest after five consecutive days of stimulation, and a total of 10 sessions. Most of the previous HD-tDCS studies have shown effective cortical stimulation and inhibition with 2.0 mA. However, several other factors such as the age of the subject and subject-specific skull thickness could have also played a role in the differing outcomes in addition to the current intensity (39). Referring to previous studies, such as that of Breitling et al. (22), on stimulated subjects with ADHD aged 10–16 years with HD-tDCS of 0.25 and 0.5 mA, considering that the subjects are children and adolescents and the skull thickness is different from that of adults, the current intensity is selected as 1.0 mA in this study. The sham group received sham stimulation, in which the subjects of the HD-tDCS group underwent under the same electrode setting. During the stimulation, the current was increased for 30 s, and after reaching 1.0 mA, the current was reduced to 0 in the following 30 s to simulate the skin feeling during HD-tDCS and make the subjects have the same subjective feeling as the real stimulation.

### Clinical symptom assessment for ADHD

#### Swanson, Nolan, and Pelham-IV rating scale

The Swanson, Nolan, and Pelham-IV rating scale (SNAP-IV rating scale) rating scale has good reliability and validity (40, 41). This scale is compiled according to the DSM-IV diagnostic criteria for ADHD, with a total of 18 items that are summarized in two factors: attention deficit (items 1–9) and hyperactivity/impulsivity (items 10–18) were scored on a scale of 4 for symptom severity (none at all 0; A little bit is one point; Not too little is 2 points; and Very many are 3 points), selected by parents according to their children's general impression. The scores are on average.

#### Conners child behavior scale parent symptom questionnaire

The Conners' Parent Rating Scales (CPRS) revised in 1978 has a total of 48 items (42). Previous research has demonstrated that the Parent Symptom Questionnaire (PSQ) has good reliability in China (Cronbach's α = 0.93) and may be used to evaluate Chinese children (43). These include the Conduct factor (items 2, 8, 14, 19, 20, 21, 22, 23, 27, 33, 34, and 39), Learning factor (items 10, 25, 31, and 37), Physical and mental factor (items 32, 41, 43, 44, and 48), Hyperactivity-impulsivity index (items 4, 5, 11, and 13), Anxiety factor (items 12, 16, 24, and 27), and Hyperactivity index (items 4, 7, 11, 13, 14, 25, 31, 33, 37, and 38). Each item was rated on a scale of 4; 0 to 3 by parents based on observation. The score was on average.

### Cognitive tasks

#### Integrated Visual and Auditory Continuous Performance Test

The IVA + CPT is a valuable tool for assessing ADHD (44, 45). The first part of the test is the Visual Attention Test. The visual stimulus numbers 0–9 were presented on the computer monitor screen, and each time, the 10 numbers were randomly arranged on the screen for about 2 s (the time was not fixed to exclude the interference of the subjects' preparatory actions); the subjects were required to find out the specific number (such as “3”) then and click the left mouse button to confirm. The test cycle was 12 min. The second part was the Auditory Attention Test: auditory stimulus numbers 0–9 were played by a computer speaker and a random number was played each time with an interval of about 2 s. The subjects were asked to identify whether the number they heard was a specific number (such as “5”) and then click the left mouse button to confirm. The test was repeated several times and lasted for 12 min. The third part was the Combination of the Audiovisual Attention Tests: whenever a random number 0–9 is displayed on the computer display screen, random numbers 0–9 were displayed on the computer speaker, and when the number displayed on the screen matches the number played by the speaker, the subject was required to confirm by clicking the left mouse button. The test interval was about 2 s and the cycle lasted for 12 min. The evaluation indices included the correct response numbers, the commission errors (false response numbers), the omissions (missed report numbers), and the average reaction time (ms) of the combination of visual, auditory, and audiovisual indices.

#### Stroop Color and Word Test

This test is a measurement paradigm of interference suppression (46), which is divided into the basic reading part: the Word Test and the Color Test; interference with the reading part: the Word Meaning Interference Test and the Color Meaning Interference Test. In the word test, the subjects are required to read out different characters on the card, including 30 Chinese characters “red, green, blue, and yellow” printed in black on a white background in 3 rows × 10 columns. In the color test, the subjects are required to read out the colors of different color blocks. There are 30 color blocks randomly arranged in 3 rows × 10 columns of “red, green, blue, and yellow.” The time (s) of completing the Word and Color Tests are recorded. The Word and Color Tests are automated processes that assess short-term attention and reading speed. In the interference with the reading part, 12 rows × 9 columns are randomly arranged in four colors: red, green, blue, and yellow, showing four kinds of Chinese characters: “red, green, blue, and yellow.” A total of 50% of the characters have the same meaning and color and 50% of the characters do not. In the Word Meaning Interference Test, if there is a color word with inconsistent meaning and color, the subjects are required to read the color instead of the Chinese character (for example, “red” is printed in green and “green” is read instead of “red”) and name the color. In the Color Meaning Interference test, the subjects are required to read the color words with inconsistent meanings and the color according to the meaning of the word and eliminate the color interference. In the Test order, after the Word Test, namely the establishment of the word dominance response, the Word Meaning Interference Test was conducted. In the same way, after the completion of the Color Test, the color dominance response was established before the Color Meaning Interference Test. The subjects were required to complete the above test quickly within the specified time (2 min). Finally, the RT (reaction time) of the interference effect of Word and Color was calculated: the RT incongruent of color and word—RT congruent of color and word.

#### The Tower of Hanoi

The mission consists of three vertical wooden poles and a fixed number of disks of different sizes (four disks in this study) with holes in them such that they can be placed on the poles. The goal is to move the disks from a starting position to a target position and arrange them in a pyramid form on the target position (47). Constraint conditions: only one disk can be moved at a time and a larger disk cannot be on top of a smaller disk to complete the task in the process. The disk must either be in the process of moving or on the pole. The image of the disk was displayed on a screen and the subject could move the disk by pressing the corresponding key on a keyboard. The evaluation included the total completion time(s) and the steps taken between the first and last moves.

The cognitive tasks above were completed on the computer with software from Nanjing Vishee Medical Technology Co., Ltd.

### Statistical analysis

SPSS 28.0 statistical software was used for data analyses. The measurement scale-data satisfying the normal distribution and homogeneity of variance were expressed as mean ± standard deviation (*M* ± SD). The nominal-data measurements were expressed as number ± percentage [*N* (%)]. The independent sample *T*-test/Chi-square test and the repeated-measures ANOVA were adopted. Then, Mauchly's sphericity test was used to evaluate the sphericity of the data before implementing the repeated measures ANOVA. If a *P*-value was > 0.05, it indicates that Mauchly's sphericity test was violated and therefore the Greenhouse–Geisser test was performed. If a *P*-value was < 0.05, it indicates that Mauchly's sphericity test was accepted and therefore Roy's largest root exact test was performed. To test the effects of HD-tDCS, repeated measures ANOVA was performed for the within-subject factor of TIME (T0, T1, T2, and T3), the between-subject factor of CONDITION (the real stimulation and the sham stimulation), and the interaction factor of TIME × CONDITION and the Bonferroni correction test as well as the Bonferroni *post-hoc* test was used. Because ANOVA has 16 variables, after Bonferroni's correction test, test statistics with a *P*-value of <0.0031 indicate significant results. It means that if the *P* value is less than alpha 0.31%, then we reject the null hypothesis and consider the result to be statistically significant. GraphPad Prism 9 was used for the diagram.

## Results

### General demographic information

During the implementation of the experiment, nine cases were lost due to “inconvenient medical treatment, busy study, troublesome treatment and evaluation process, uncomfortable stimulation of the head, and no treatment effect,” including four cases in the HD-tDCS group and five cases in the Sham group, which were not included in the statistics. Before the experiment, the subjects were grouped and divided. Finally, 47 subjects completed the experiment and entered the stage of result analysis (Figure 1). There were 24 subjects in the HD-tDCS group, including 14 boys and 10 girls, and their average age was (11.29 ± 2.51) years. There were 23 subjects in the Sham group, including 13 boys and 10 girls; the average age was (11.74 ± 2.59) years. There were no significant differences between the two groups in terms of age, gender, total IQ, educational years, whether they come from a single-parent family, age of onset, course of disease, and ADHD type between the two groups (*P* > 0.05). In addition, SNAP-IV and PSQ were taken as baseline clinical manifestations, and there was no significant difference between the two groups (Table 1).

**Table 1:** Subject's demographic characteristics, intelligence, and clinical manifestations.

**Variables**	**HD-tDCS**	**Sham**	***T*/χ^2^ (*P*)**
Number	24	23	–
Age (*M* ± SD)	11.29 ± 2.51	11.74 ± 2.59	−0.601 (0.551)
Gender (%boys)	14 (58.33%)	13 (56.52%)	0.016 (0.900)
Total IQ (*M* ± SD)	90.50 ± 5.073	89.39 ± 3.577	0.862 (0.393)
Educational years (*M* ± SD)	4.15 ± 2.119	4.91 ± 2.521	−1.131 (0.264)
Whether comes from a single-parent family (%yes)	8 (33.3%)	5 (21.7%)	1.945 (0.378)
Age of onset (*M* ± SD)	9.17 ± 2.220	9.37 ± 2.024	−0.327 (0.745)
Course of disease (*M* ± SD)	2.125 ± 1.279	2.370 ± 1.693	−0.560 (0.578)
ADHD type			
Combined type (%)	17 (70.8%)	17 (73.9%)	0.122 (0.941)
Inattentive type (%)	3 (12.5%)	3 (13.0%)	
Hyperactive impulsive type (%)	4 (16.7%)	3 (13.0%)	
SNAP-IV (*M* ± SD)			
Attention deficit factor	2.217 ± 0.380	2.198 ± 0.330	0.181 (0.858)
Hyperactivity/impulsivity factor	2.120 ± 0.271	2.209 ± 0.211	−1.245 (0.220)
PSQ (*M* ± SD)			
Conduct factor	1.614 ± 0.345	1.528 ± 0.355	0.837 (0.407)
Learning factor	1.677 ± 0.486	1.804 ± 0.532	−0.856 (0.397)
Physical and mental factor	1.741 ± 0.384	1.947 ± 0.396	−1.810 (0.077)
Hyperactivity-impulsivity index	1.802 ± 0.312	1.793 ± 0.366	0.087 (0.931)
Anxiety factor	1.677 ± 0.308	1.684 ± 0.370	−0.078 (0.939)
Hyperactivity index	1.629 ± 0.428	1.747 ± 0.378	−1.004 (0.321)

### Clinical symptom assessment results from SNAP-IV and PSQ

Repeated measurement ANOVA was performed for the SNAP-IV and PSQ scores of both groups. The results showed no statistical significance (*P* > 0.05) in terms of TIME, CONDITION, and interaction TIME×CONDITION, suggesting that the Attention Deficit and Hyperactivity/Impulsivity factor scores of SNAP-IV, Conduct factor, Learning factor, Physical and mental factor, Hyperactivity-impulsivity index, Anxiety factor, and the Hyperactivity index scores of PSQ did not change with the intervention time in the HD-tDCS group and the Sham tDCS group.

### Comparison of cognitive task results

**Figure 4:**
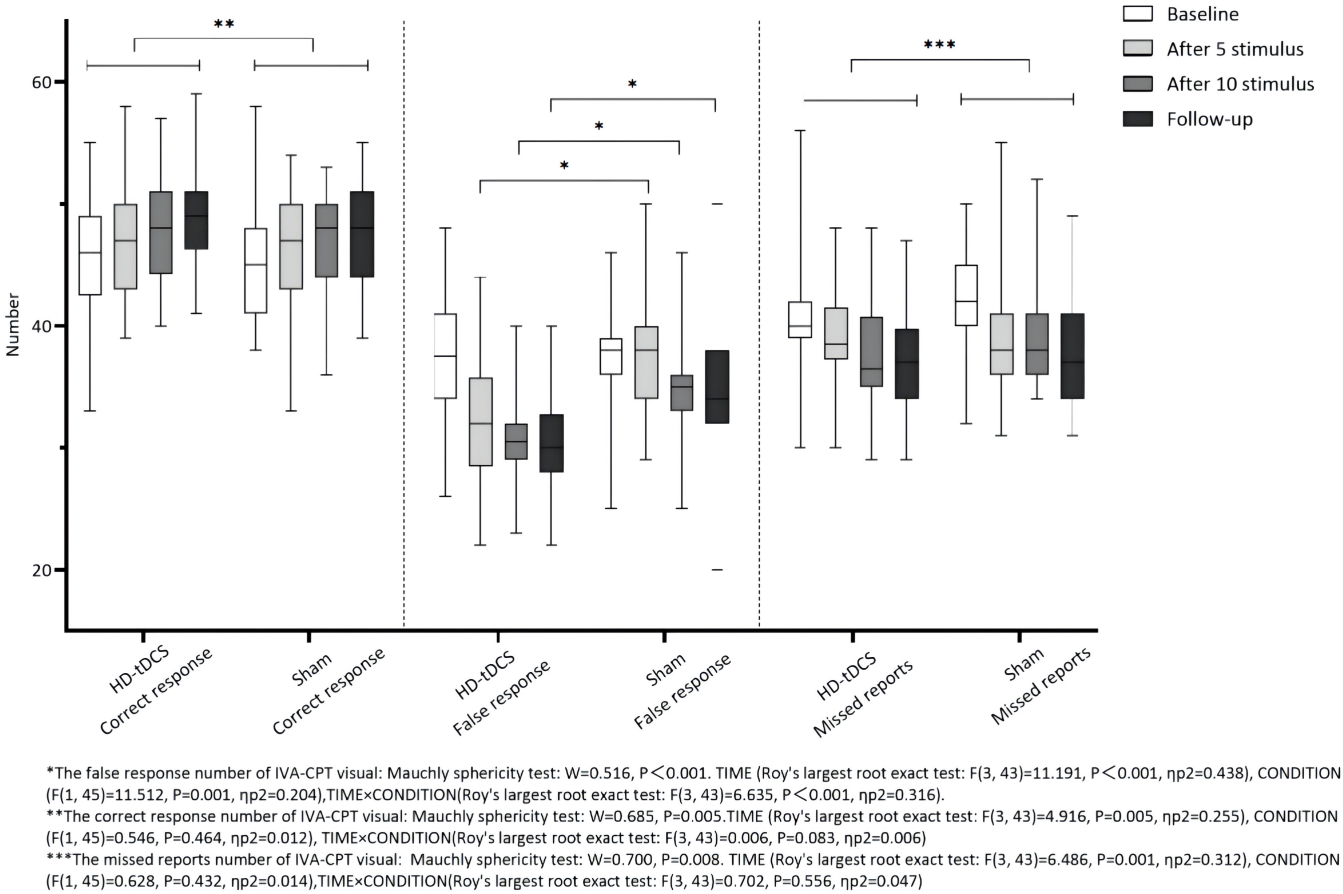
Comparison of IVA-CPT visual between the two groups at different time points. *The commission errors of IVA-CPT visual: Mauchly sphericity test: *W* = 0.516, *P* < 0.001 TIME (Roy's largest root exact test: *F*_(3,43)_ = 11.191, *P* < 0.001, ηp2 = 0 438), CONDITION (*F*_(1,45)_ = 11.512, *P* = 0.001, ηp2 = 0.204), TIME × CONDITION(Roy's largest root exact test: *F*_(3,43)_ = 6.635, *P* = 0.001, ηp2 = 0.316). **The correct response number of IVA-CPT visual: Mauchly sphericity test: *W* = 0.685, *P* = 0.005. TIME (Roy's largest root exact test: *F*_(3,43)_ = 4.916, *P* = 0.005, ηp2 = 0.255), CONDITION (*F*_(1,45)_ = 0.546, *P* = 0.464, ηp2 = 0.012), TIME × CONDITION (Roy's largest root exact test: *F*_(3,43)_ = 0.006, *P* = 0.083, ηp2 = 0.006). ***The omission of IVA-CPT visual: Mauchly sphericity test: *W* = 0.700, *P* = 0.008. TIME (Roy's largest root exact test: *F*_(3,43)_ = 6,486, *P* = 0.001, ηp2 = 0.312), CONDITION (*F*_(1,45)_ = 0.628, *P* = 0.432, ηp2 = 0.014), TIME × CONDITION (Roy's largest root exact test: *F*_(3,43)_ = 0.702, *P* = 0.556, ηp2 = 0.047).

Repeated measurement ANOVA was performed for the correct response number of visual IVA-CPT. There were no statistically significant main effects in terms of TIME (*F*_(3,43)_ = 4.916, *P* = 0.005), CONDITION (*F*_(1,45)_ = 0.546, *P* = 0.464), and interaction effect for TIME × CONDITION (*F*_(3,43)_ = 0.006, *P* = 0.083). Repeated measurement ANOVA was performed for the commission errors of visual IVA-CPT. There were statistically significant main effects for TIME (*F*_(3,43)_ =11.191, *P* < 0.001), CONDITION (*F*_(1,45)_ = 11.512, *P* = 0.001), and interaction effect for TIME×CONDITION (*F*_(3,43)_ = 6.635, *P* < 0.001). A Bonferroni correction test (*post-hoc*) showed the false response number decreased under the HD-tDCS condition when compared with T0 and T1 (*P* < 0.001), T2 (*P* < 0.001), and T3 (*P* < 0.001). Repeated measurement ANOVA was performed for the omission of visual IVA-CPT. There were statistically significant main effects for TIME (*F*_(3,43)_ = 6.486, *P* = 0.001), but no statistically significant main effect for the CONDITION effect (*F*_(1,45)_ = 0.628, *P* = 0.432) and the interaction effect for TIMEർONDITION (*F*_(3,43)_ = 0.702, *P* = 0.556; Figure 4).

**Figure 5:**
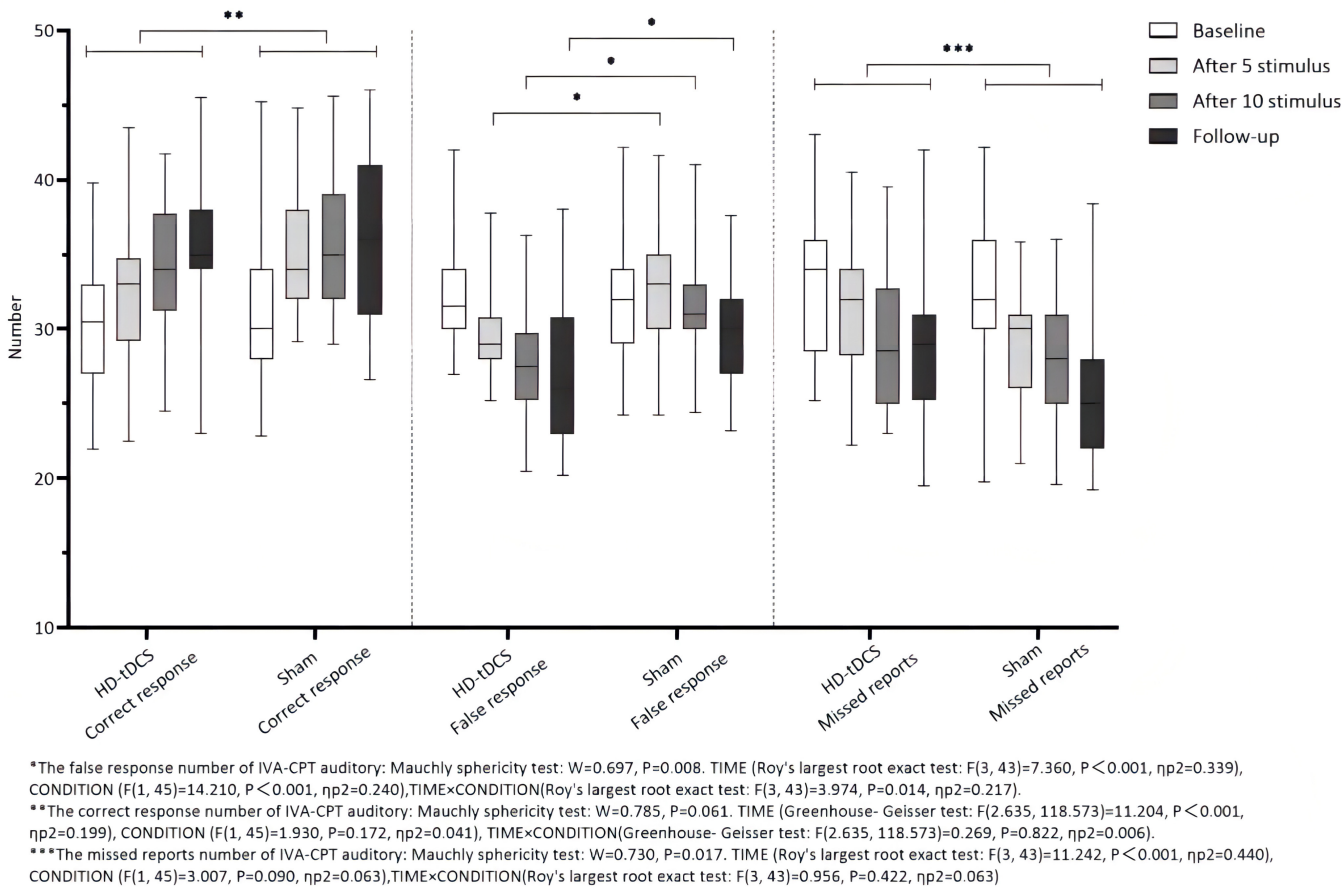
Comparison of IVA-CPT auditory between the two groups at different time points. *The commission errors of IVA-CPT auditory: Mauchly sphericity test: *W* = 0.697, *P* = 0.008. TIME (Roy's largest root exact test: *F*_(3,43)=_ 7.360, *P* < 0.001, ηp2 = 0.339), CONDITION (*F*_(1,45)_ = 14.210, *P* < 0.001, ηp2 = 0.240), TIME × CONDITION (Roy's largest root exact test: *F*_(3,43)_ = 3.974, *P* = 0.014, ηp2 = 0.217). **The correct response number of IVA-CPT auditory: Mauchly sphericity test: *W* = 0.785, *P* = 0.061. TIME (Greenhouse-Geisser test: *F*_(2.635,118.573)_ = 11.204, *P* < 0.001, ηp2 = 0.199), CONDITION (*F*_(1,45)_ = 1.930, *P* = 0.172, ηp2 = 0.041), TIME × CONDITION (Greenhouse-Geisser test: *F*_(2.635,118.573)_ = 0.269, *P* = 0.822, ηp2 = 0.006). ***The omission of IVA-CPT auditory: Mauchly sphericity test: *W* = 0.730, *P* = 0.017. TIME (Roy's largest root exact test: *F*_(3,43)_ = 11.242, *P* = 0.001, ηp2 = 0.440), CONDITION (*F*_(1,45)_ = 3.007, *P* = 0.090, ηp2 = 0.063), TIME × CONDITION (Roy's largest root exact test: *F*_(3,43)_ = 0.956, *P* = 0.422, ηp2 = 0.063).

Repeated measurement ANOVA was performed for the correct response number of auditory IVA-CPT. There were statistically significant main effects in terms of TIME (*F*_(2.635,118.573)_ = 11.204, *P* < 0.001), but no statistically significant effect for CONDITION (*F*_(1,45)_ = 1.930, *P* = 0.172) and the interaction effect for TIME × CONDITION (*F*_(2.635,118.573)_ = 0.269, *P* = 0.822). Repeated measurement ANOVA was performed for the commission errors of auditory IVA-CPT. There were statistically significant main effects for TIME (*F*_(3,43)_ = 7.360, *P* < 0.001) and CONDITION (*F*_(1,45)_ = 14.210, *P* < 0.001), but no statistically significant interaction effect for TIME×CONDITION (*F*_(3,43)_ = 3.974, *P* = 0.014). Repeated measurement ANOVA was performed for the omission of auditory IVA-CPT. There were statistically significant main effects for TIME (*F*_(3,43)_ = 11.242, *P* < 0.001), but no statistically significant main effects for CONDITION (*F*_(1,45)_ = 3.007, *P* = 0.090) and the interaction effect for TIME × CONDITION (*F*_(3,43)_ = 0.956, *P* = 0.422; Figure 5).

**Figure 6:**
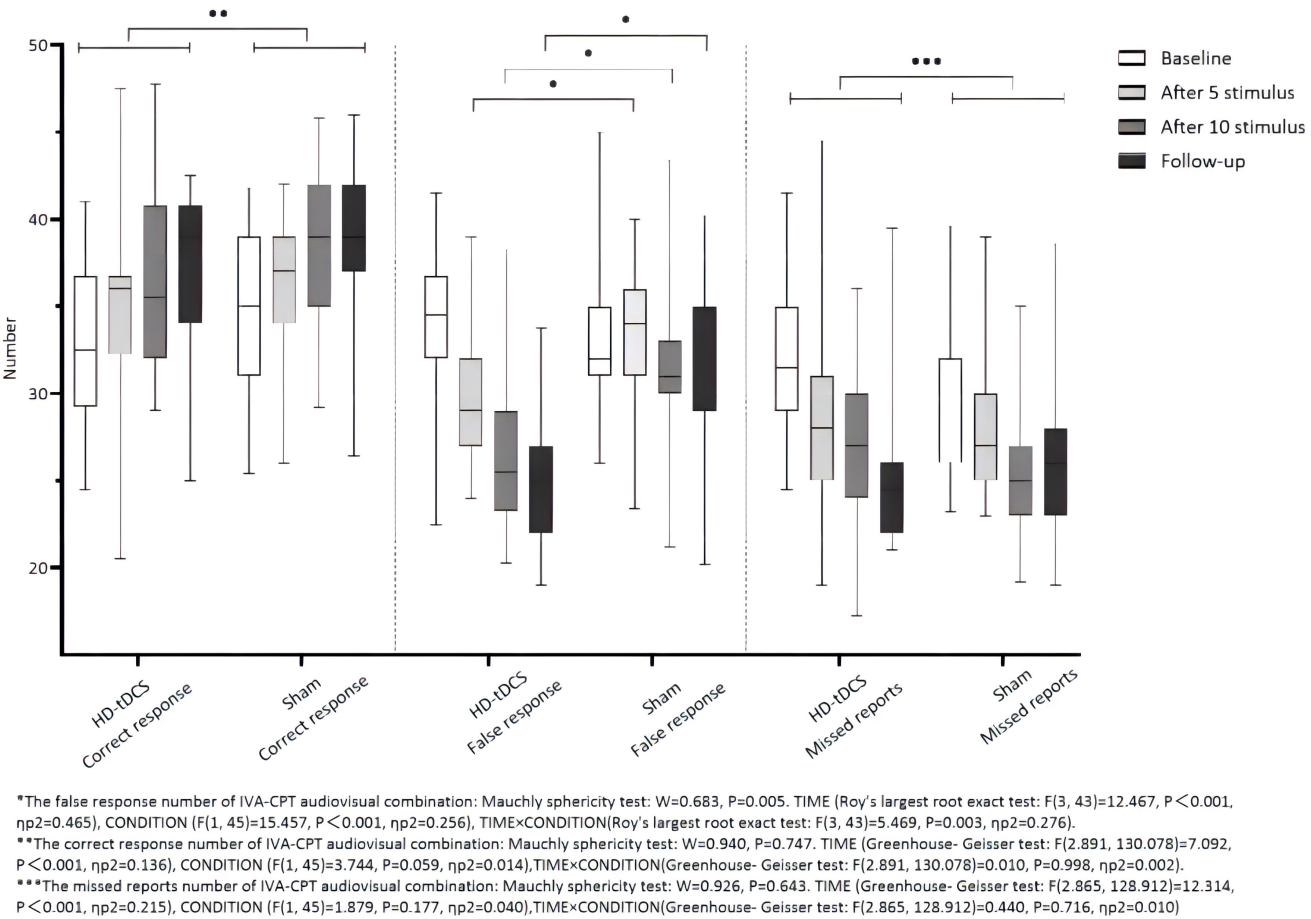
Comparison of IVA-CPT audiovisual combination between the two groups at different time points. *The commission errors of IVA-CPT audiovisual combination: Mauchly sphericity test: *W* = 0.683, *P* = 0.005. TIME (Roy's largest root exact test: *F*_(3,43)=_ 12.467, *P* < 0.001, ηp2 = 0.465), CONDITION (*F*_(1,45)_ = 15.457, *P* < 0.001, ηp2 = 0.256), TIME × CONDITION (Roy's largest root exact test: *F*_(3,43)_ = 5.469, *P* < 0.003, ηp2 = 0.276). **The correct response number of IVA-CPT audiovisual combination: Mauchly sphericity test: *W* = 0.940, *P* = 0.747. TIME (Greenhouse-Geisser test: *F*_(2.891,130.078)_ = 7.092, *P* < 0.001, ηp2 = 0.136), CONDITION (*F*_(1,45)_ = 3.744, *P* = 0.059, ηp2 = 0.014), TIME × CONDITION (Greenhouse-Geisser test: *F*_(2.891,130.078)_ = 0.010, *P* = 0.998, ηp2 = 0.002). ***The omission of IVA-CPT audiovisual combination: Mauchly sphericity test: *W* = 0.926, *P* = 0.643. TIME (Greenhouse-Geisser test: *F*_(2.865,128.912)_ = 12.314, *P* = 0.001, ηp2 = 0.215), CONDITION (*F*_(1,45)_ = 1.879, *P* = 0.177, ηp2 = 0.040), TIME × CONDITION (Greenhouse-Geisser test: *F*_(2.865,128.912)_ = 0.440, *P* = 0.716, ηp2 = 0.010).

Repeated measurement ANOVA was performed for the correct response number of the audiovisual combination of IVA-CPT. There were statistically significant main effects for TIME (*F*_(2.891,130.078)_=7.092, *P* < 0.001), but no statistically significant main effect for CONDITION (*F*_(1,45)_=3.744, *P* = 0.059) and the interaction effect for TIME×CONDITION (*F*_(2.891,130.078)_ = 0.010, *P* = 0.998). Repeated measurement ANOVA was performed for the commission errors of the audiovisual combination IVA-CPT. There were statistically significant main effects for TIME (*F*_(3,43)_ =12.467, *P* < 0.001), CONDITION (*F*_(1,45)_ =15.457, *P* < 0.001) and the interaction effect for TIME × CONDITION (*F*_(3,43)_ =5.469, *P* = 0.003). A Bonferroni correction test (*post hoc*) showed a decrease in the false response number in the HD-tDCS condition compared with T0 and T1 (*P* < 0.001), T2 (*P* < 0.001), and T3 (*P* < 0.001). Repeated measurement ANOVA was performed for the omission of the audiovisual combination IVA-CPT. There were statistically significant main effects for TIME (*F*_(2.865,128.912)_ = 12.314, *P* < 0.001), but no statistically significant main effect for CONDITION (*F*_(1,45)_ = 1.879, *P* = 0.177) and the interaction effect for TIME × CONDITION (*F*_(2.865,128.912)_ = 0.440, *P* = 0.716; Figure 6).

**Figure 7:**
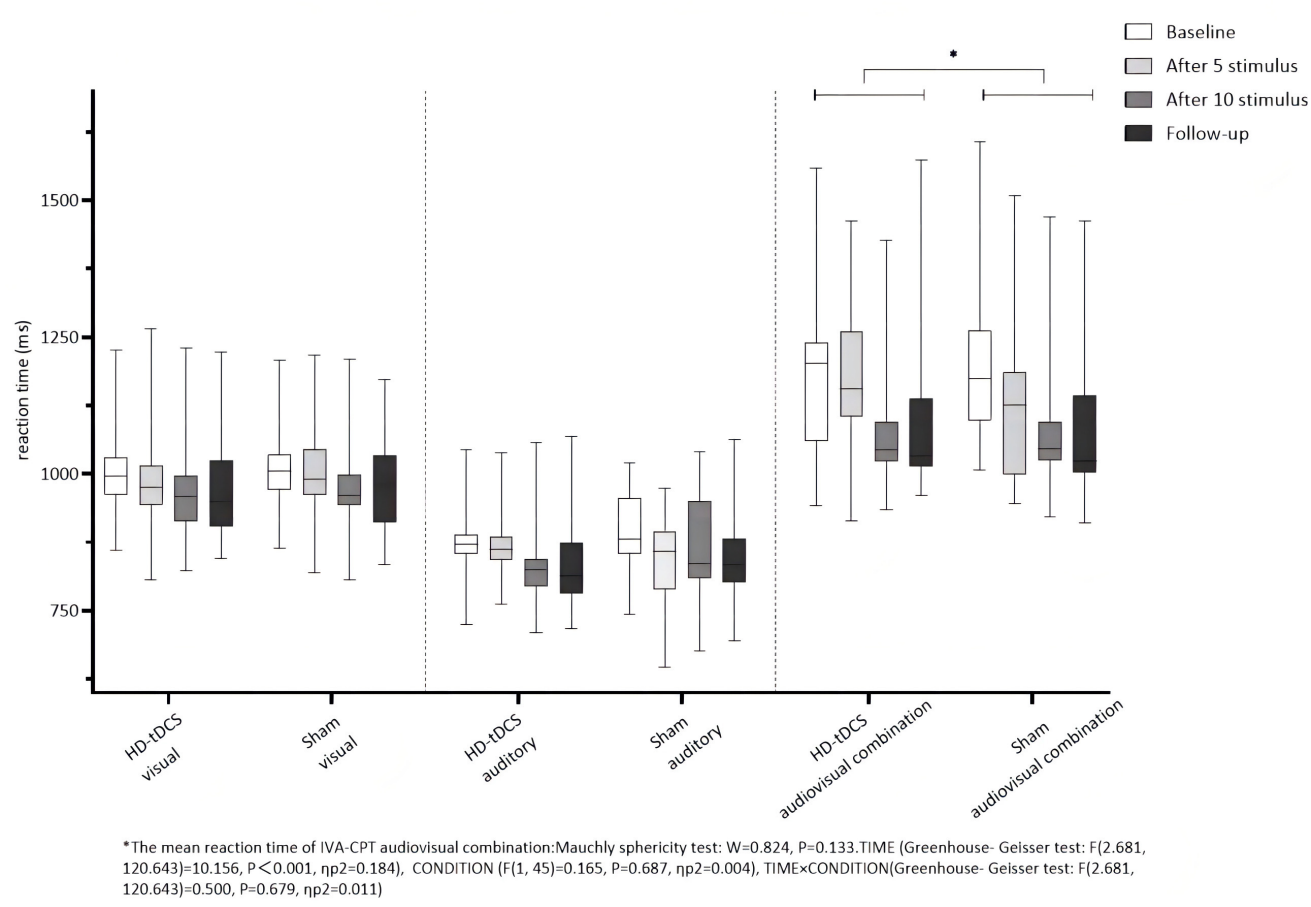
Comparison of IVA-CPT mean reaction time between the two groups at different time points. *The mean reaction time of IVA-CPT audiovisual combination: Mauchly sphericity test: *W* = 0.824, *P* = 0.133. TIMF (Greenhouse-Geisser test: *F*_(2.681,120.643)_ = 10.156, *P* < 0.001, ηp2 = 0.184), CONDITION (*F*_(1,45)_ = 0.165, *P* = 0.687, np2 = 0.004), TIME × CONDITION (Greenhouse-Geisser test: *F*_(2.681,120.643)_ = 0.500, *P* = 0.679, ηp2 = 0.011).

Repeated measurement ANOVA was performed for the mean reaction time of visual IVA-CPT. There were no statistically significant main effects for TIME (*F*_(2.877,129.444)_ =1.739, *P* = 0.162), CONDITION (*F*_(1,45)_ =0.471, *P* = 0.496) and the interaction effect for TIME × CONDITION (*F*_(2.877,129.444)_ = 0.220, *P* = 0.875). Repeated measurement ANOVA was performed for the mean reaction time of auditory IVA-CPT. There were no statistically significant main effects for TIME (*F*_(2.878,129.494)_ = 2.002, *P* = 0.117), CONDITION (*F*_(1,45)_ = 0.080, *P* = 0.778) and the interaction effect for TIME × CONDITION (*F*_(2.878,129.494)_ = 0.574, *P* = 0.626). Repeated measurement ANOVA was performed for the mean reaction time of the audiovisual combination IVA-CPT. There were statistically significant main effects for TIME (*F*_(2.681,120.643)_ = 10.156, *P* < 0.001), but no statistically significant main effect for CONDITION (*F*_(1,45)_ = 0.165, *P* = 0.687) and the interaction effect for TIME × CONDITION (*F*_(2.681,120.643)_ = 0.500, *P* = 0.679; Figure 7).

These results suggested that the correct response number of the visual, the mean visual, and the auditory reaction time of both groups did not change with the intervention time, the correct response number of auditory and audiovisual combination, the commission errors of auditory reaction time, the omission of the visual, auditory, and audiovisual combination, and the average reaction time for the audiovisual combination that gradually increased or decreased with the intervention time in both groups, but there was no significant increase or decrease in the HD-tDCS group than in the Sham tDCS group and the commission errors of visual and audiovisual combination in both groups decreased gradually with the intervention time. Furthermore, the HD-tDCS group compared with the Sham tDCS group was more significantly decreased after the 5th intervention, the 10th intervention, and the 6th-week follow-up.

**Figure 8:**
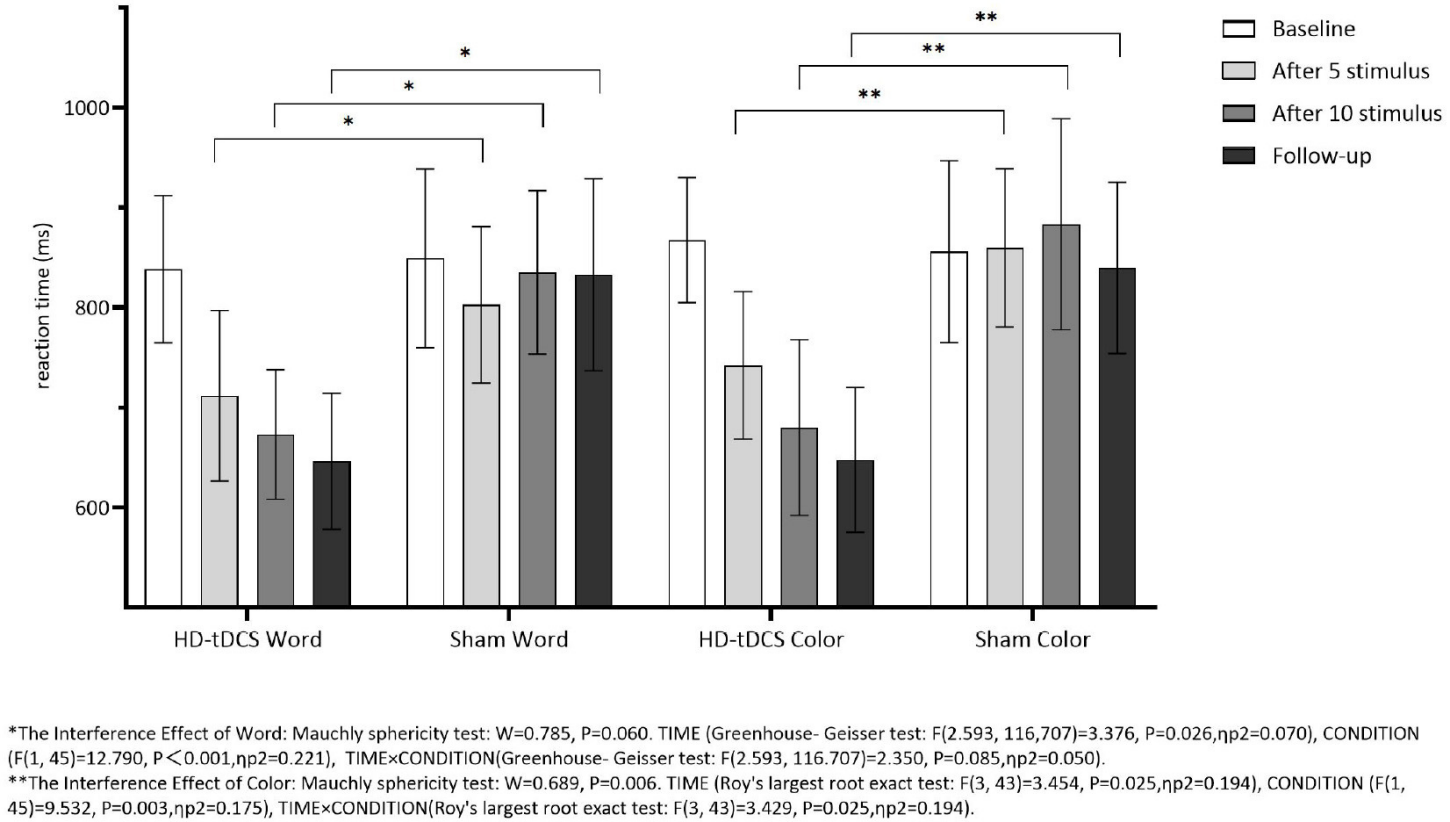
Comparison of Stoop Interference effect RT of Word and Color between the two group at different time. *The interference effect of word: Mauchly sphericity test: *W* = 0.785, *P* = 0.060. TIME (Greenhouse-Geisser test: *F*_(2.593,116.707)_ = 3.376, *P* = 0.026, ηp2 = 0.070), CONDITION (*F*_(1,45)_ = 12.790, *P* = 0.001, ηp2 = 0.221), TIME × CONDITION (Greenhouse-Geisser test: *F*_(2.593,116.707)_ =2.350, *P* = 0.085, ηp2 = 0.050). **The interference effect of color: Mauchly sphericity test: *W* = 0.689, *P* = 0.006. TIME (Roy's largest root exact test: *F*_(3,43)_ = 3.454, *P* = 0.025, ηp2 = 0.194), CONDITION (*F*_(1,45)_ = 9.532, *P* < 0.003, ηp2 = 0.175), TIME × CONDITION (Roy's largest root exact test: *F*_(3,43)_ = 3.429, *P* = 0.025, ηp2 = 0.194).

Repeated measurement ANOVA was performed for the Interference RT of Word. There was no statistically significant main effect for TIME (*F*_(2.593,116,707)_ = 3.376, *P* = 0.026) and the interaction effect for TIME × CONDITION (*F*_(2.593,116.707)_ = 2.350, *P* = 0.085). Repeated measurement ANOVA was performed for the Interference RT of Color. There were no statistically significant main effects for TIME (*F*_(3,43)_ = 3.454, *P* = 0.025) and the interaction effect for TIME × CONDITION (*F*_(3,43)_ = 3.429, *P* = 0.025). Figure 8 indicates that the Interference RT of Color and Word in both groups did not change with the intervention time.

**Figure 9:**
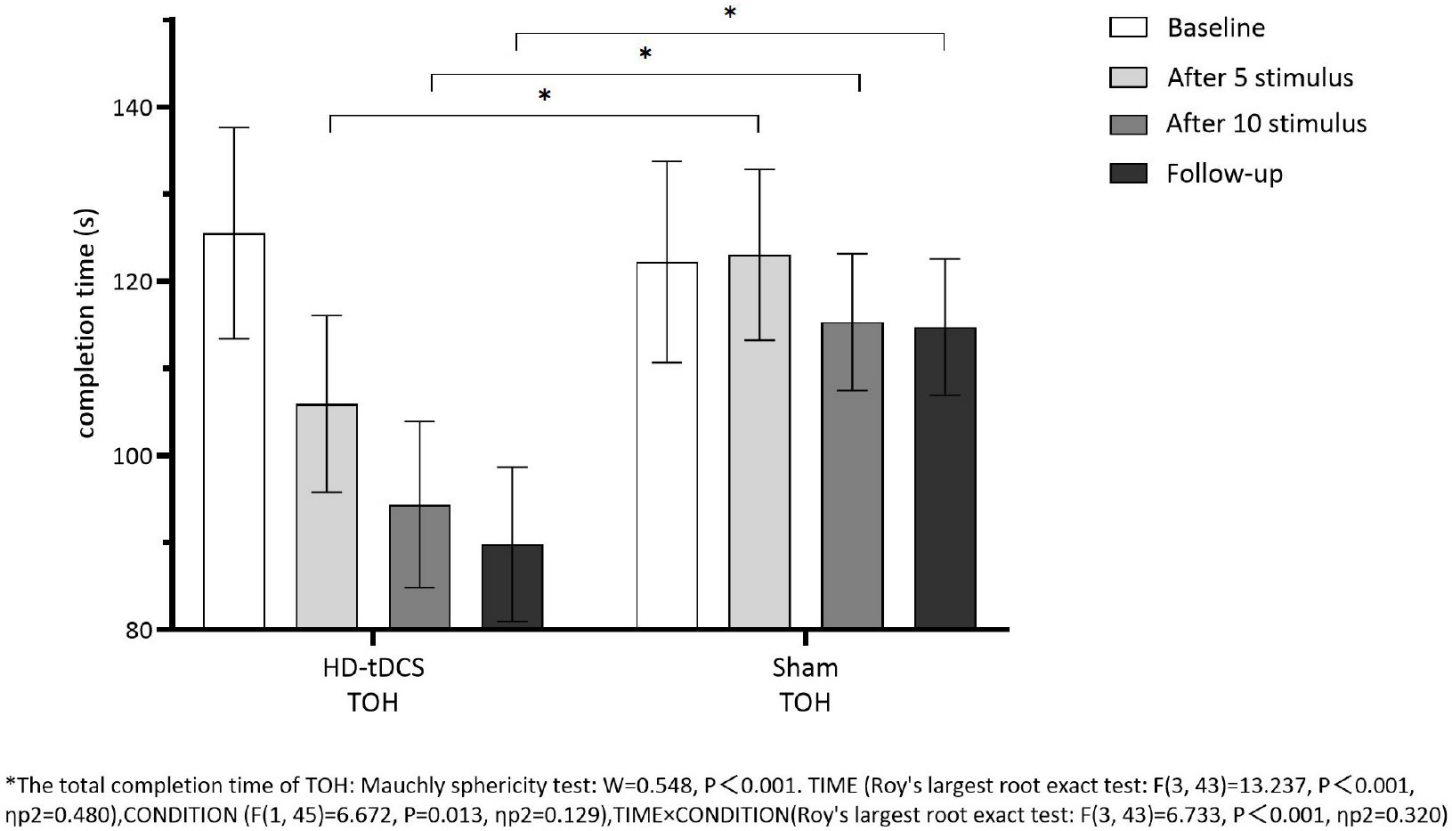
Comparison of TOH completion time between the two groups at different time. *The total completion time oh TOH: Mauchly sphericity test: *W* = 0.548, *P* < 0.001. TIME (Roy's largest root exact test: *F*_(3,43)_ = 13.237, *P* < 0.001, ηp2 = 0.480), CONDITION (*F*_(1,45)_ = 6.672, *P* = 0.013, ηp2 = 0.129), TIME × CONDITION (Roy's largest root exact test: *F*_(3,43)_ = 6.733, *P* < 0.001, ηp2 = 0.320.

**Figure 10:**
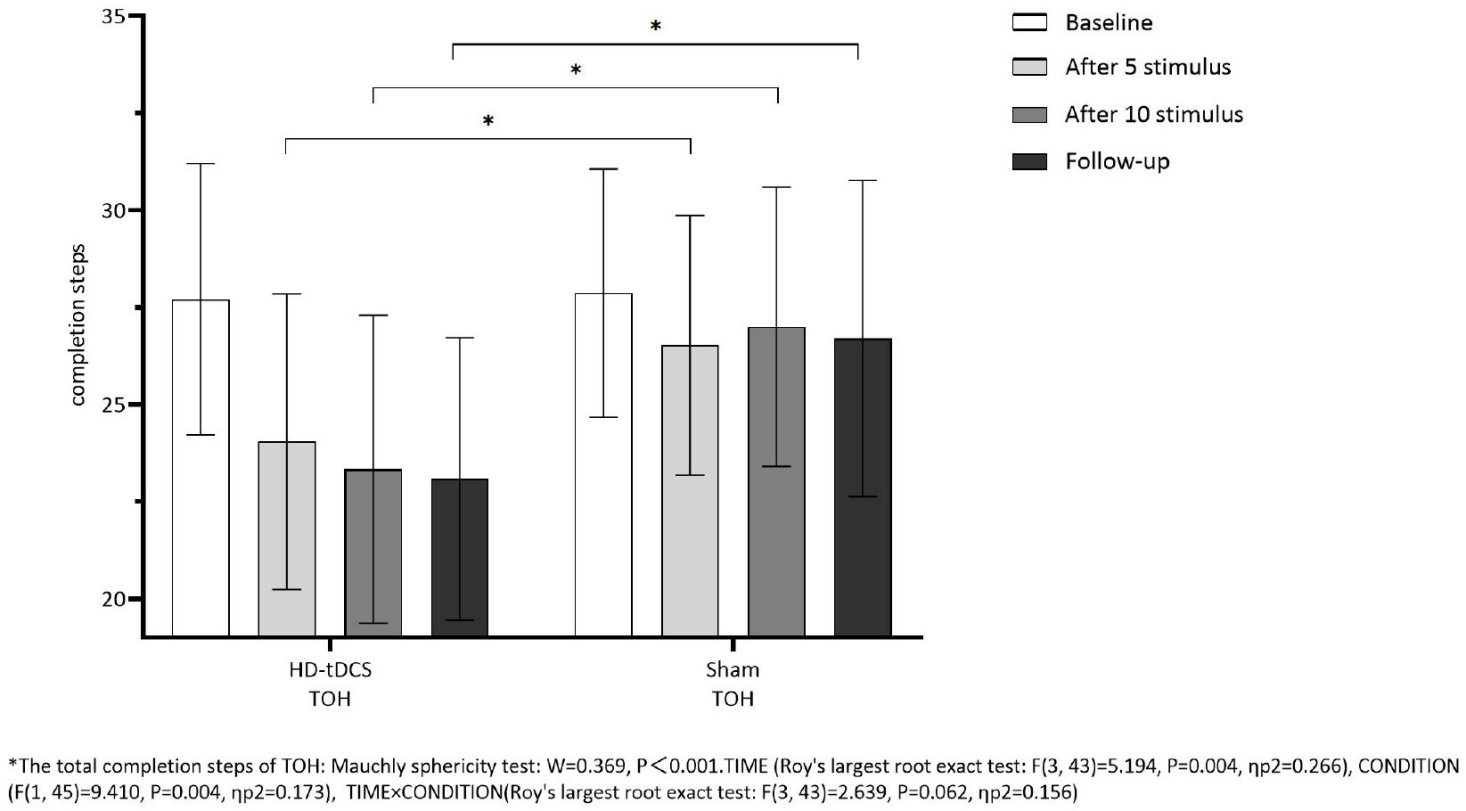
Comparison of TOH completion steps between the two groups at different time. *The total completion steps of TOH: Mauehly sphericity test: *W* = 0.369, *P* < 0.001. TIME (Roy's largest root exact test: *F*_(3,43)_ = 5.194, *P* = 0.004, ηp2 = 0.266), CONDITION (*F*_(1,45)_ = 9.410, *P* = 0.004, ηp2 =0.173), TIME × CONDITION (Roy's largest root exact test: *F*_(3,43)_ = 2.639, *P* = 0.062, ηp2 = 0.156).

Repeated measurement ANOVA was performed for the total completion time of TOH. There were statistically significant main effects for TIME (*F*_(3,43)_ =13.237, *P* < 0.001) and the interaction effect for TIME × CONDITION (*F*_(3,43)_ =6.733, *P* < 0.001). A Bonferroni correction test (*post hoc*) showed that the total completion time reduced in the HD-tDCS condition compared to T0 and T1 (*P* < 0.001), T2 (*P* < 0.001), and T3 (*P* < 0.001). Repeated measurement ANOVA was performed for the total completion steps of TOH. There were no statistically significant main effects for TIME (*F*_(3,43)_ = 5.194, *P* = 0.004), CONDITION (*F*_(1,45)_ = 9.410, *P* = 0.004) and the interaction effect for TIME × CONDITION (*F*_(3,43)_ = 2.639, *P* = 0.062). It suggests that the total completion steps of TOH in both groups did not change with the intervention time, but the total completion time of TOH in both groups decreased gradually with the intervention time. Furthermore, the HD-tDCS group compared with the Sham tDCS group was more significantly reduced after the 5th intervention, the 10th intervention, and the 6th-week follow-up (Figures 9, 10).

## Discussion

This study observed some positive effects. The results showed that the commission errors of the visual and audiovisual combination of IVA-CPT tasks changed significantly in the two groups. Furthermore, comparing real stimulation with sham tDCS, there was a significant improvement in the commission errors after real HD-tDCS intervention, and this effect was even reflected in the follow-up 6 weeks later. The IVA-CPT tasks were not only attention-maintaining tasks but also inhibition-control tasks. The tasks were intended to be mildly boring to produce the omission (i.e., inattention) and commission errors (i.e., impulsivity) through a series of trial sets requiring responding and not responding, respectively. The commission errors of IVA-CPT reflected the inhibition ability of impulse. The subjects had to suppress impulses instead of making mistakes when they received the visual and audiovisual combination stimuli (44, 45, 48). Therefore, it showed that real HD-tDCS can improve the inhibitory control of the subjects in addition to improving attention maintenance. This was similar to previous studies that showed that tDCS tended to improve significantly only in interference control and inhibition, but not in working memory or reaction time variability in the analyses of neuropsychological performance measures (49). This effect is also supported by studies on the mechanism of tDCS, which regulates the concentration levels of the excitatory neurotransmitter glutamate and the inhibitory neurotransmitter γ-aminobutyric acid (GABA). A magnetic resonance spectrum study found that GABA concentration increased after ATDCS stimulation, while glutamate concentration decreased after CTDCS stimulation (50). Some researchers believe that part of the mechanism of tDCS is to regulate the excitatory and inhibitory balance of the cortex (E/I) (51). Some studies suggested that, although the improvement of inhibitory control is assumed to be caused by the enhanced activity of the stimulating region, many experiments do not stimulate the target region alone. For example, the use of large electrodes (35 cm^2^ surface area) will cause extensive changes in cortical excitability, which may lead to changes in the overall arousal level of the brain (52). Therefore, it cannot be simply identified as the therapeutic effect produced by a certain area. Sotnikova et al. used ATDCS to stimulate the dorsolateral prefrontal cortex (DLPFC) and analyzed the functional connectivity of the brain through functional magnetic resonance after stimulation. The results showed that, in the N-Back Task, whether the left DLPFC is under the action of the electrode or the left premotor cortex, the left auxiliary motor cortex, and the precuneus are not under the action of the electrode, tDCS-induced activity in these regions, suggesting that anodal tDCS can lead to increased neuronal activation and connectivity, which stimulates not only the brain regions below the electrode but also the possibly other brain regions further away (53). These studies indicate that HD-tDCS is the future direction of research. As HD-tDCS solves the problem of traditional tDCS affecting the stimulation target and the outer brain region, the stimulation current is limited to the region below the electrode, thus improving the accuracy and excluding the interference of non-target region stimulation. However, there are few studies on HD-tDCS and no studies on the right OFC. In the past, only Breitling et al. (22) studied the effect of HD-tDCS on the right IFG in 33 adolescent ADHD subjects for five consecutive days. In this study, HD-tDCS was applied to OFC, and the neuropsychological measures showed that it had a positive effect on attention maintenance and inhibitory control. It was consistent with the role of OFC in the OFCSTC loop, and it also confirmed our hypothesis that tDCS activated OFC to improve impulse control difficulties and emotional processing, thereby further improving the performance of cognitive tasks, such as attention maintenance and inhibitory control.

Another interesting result of this study was that, after the stimulation of real tDCS, the TOH completion time decreases, while the number of TOH completion steps did not change, which seemed to contradict the results of the aforementioned improved inhibitory control. The TOH is a problem that cannot be solved in one step. Subjects need to plan a reasonable sequence of steps to follow the rules and use as few steps as possible. The functions measured by TOH include cognitive planning, problem-solving, attention shifting, and attention maintenance. Inhibitory control also involved solving the TOH problem, in which subjects had to temporarily shift the smaller disk away from where it should end up to place the larger disk in the desired position. Working memory also participates in the whole process of TOH problem-solving, which is inseparable from the spatial memory of the location of the disk, which is undoubtedly a kind of working memory activity to remember the location of a specific disk while moving the disk. If impulsivity control or working memory had been improved in the TOH task, the number of TOH completed steps should have been reduced, but this was not the case. The TOH only increased the time of accomplishment, but not the number of steps required for accomplishment. The possible explanation is that real tDCS improves attention maintenance, and subjects need to increase sustained attention to complete tasks without distraction. In this study, although the time of completing TOH was significantly shortened after real HD-tDCS intervention, there were no significant differences between the two groups in the reaction time, the omission of the IVA-CPT task, and the Interference RT of Stroop Color and Word. The interference effect of Stroop Word and Color reflects attention duration, alertness, and cognitive processing speed. Meanwhile, the subjects need to suppress the automatic processing response to the word meaning or color meaning itself, eliminate the interference of the dominant stimulus attribute, respond to the inferior attribute of the stimulus, and evaluate the inhibition control ability of the subjects. This could be interpreted as that the TOH has higher and more complex difficulties than IVA-CPT and Stroop, requiring more executive function mobilization, which is consistent with previous studies. Gill et al. (54) found that ATDCS stimulation is more effective with a higher working memory load. Another possible explanation, similar to the absence of significant improvement in ADHD symptoms, is that our study may have done too few sessions to observe the effect of the Stoop effect.

In terms of clinical symptoms of ADHD, the results of this study show that there were no significant changes in the SNAP-IV and PSQ scores, and each factor of the two groups before and after the intervention indicates that HD-tDCS, despite real stimulus or sham stimulus, had no obvious immediate effect on the overall symptoms of ADHD. Most of the previous tDCS studies focused on neuropsychological changes, and only a few studies focused on clinical symptoms. Some researchers suggested that there is a dissociation between neuropsychological deficits and clinical symptoms of ADHD, which means that even if there is improvement in the neuropsychological deficits after or during tDCS, such as improvement in inhibitory control and WM, it does not mean that the clinical symptoms have improved as well (55). Meta-analyses of tDCS studies targeting mostly the dorsolateral prefrontal cortex show small effects on cognitive improvements with only two out of three studies showing clinical improvements (21). The systematic retrieval and meta-analysis of tDCS studies showed that most anode tDCS of the left dlPFC had only a very limited trend-level effect in improving inhibition and processing speed, and there was no evidence of alleviation in attention and other clinical symptoms (20). However, other researchers have come up with different conclusions. Brauer et al., by meta-analyzing 13 studies, including 20 study arms, showed that tDCS had an immediate effect on overall symptom severity, inattention, and impulsivity, but not on hyperactivity. The results were significant in children and adolescents. The follow-up data (3 days−4 weeks after stimulation) suggested an ongoing beneficial effect regarding overall symptom severity and a delayed effect on hyperactivity (49). They came to this conclusion on the basis that, although most of these studies did not provide a clinical outcome replacement for assessing the effect of tDCS on cognitive functioning in ADHD, there are several studies that report high correlations between different executive dysfunctions and ADHD core symptoms (56). Soff et al. observed that tDCS could improve the subjects' working memory and memory consolidation ability, thereby alleviating symptoms of inattention and hyperactivity, by following five consecutive anodal tDCS sessions. By the 7th day after the treatment, a long-lasting tDCS effect was implied when applying for repeated sessions (57). Previous studies showed that the tDCS physiological effects might depend on the stimulation duration and current intensity with the potential for long-lasting neuroplastic changes after multiple sessions, likely due to the changes in the synaptic strength induced by long-term potentiation (LTP)-like response and metaplasticity mechanisms (11, 58). However, this delayed effect was not observed for the 10 repeated sessions in this study and the effect of HD-tDCS after 6 weeks of follow-up was also insignificant. The likely explanation was that most of the previous studies tested five sessions and only two studies tested larger numbers of sessions. Westwood et al. (59) found no improvement after 15 sessions and Leffa et al. (17) found an improvement after 28 sessions but not after 14 sessions. This suggested that we need more sessions. Our study may have done too few sessions to observe the effect of the clinical symptoms.

In terms of other neuropsychological indicators, the results of this study showed that, compared with sham stimulus, real HD-tDCS had no significant changes in the correct response number of auditory and audiovisual combination, the commission errors of auditory, the omission of visual, auditory, and audiovisual combination, and the average reaction time of audiovisual combination. The average reaction time of IVA-CPT reflects alertness, cognitive processing speed, and hand-eye-ear coordination. The number of missing reports in IVA-CPT reflects the subjects' attention deficits, that is the intensity and stability of attention. This is partly similar to previous studies. Ouellet et al. evaluated the executive function of healthy subjects by the Iowa Gambling Task, the Stroop Task, the Visual Simulation Scale, the Continuous Work Task, and the Stop Signal Task, among others, after receiving 1.5 mA ATDCS in the left or right OFC. The results showed that subjects receiving ATDCS stimulation of the OFC had more favorable decision-making ability, but tDCS had no effect on attention level (60). The findings of the use of tDCS to improve ADHD cognition were mixed, with some positive results on improving cognition. However, the effect value observed in the meta-analysis is very small. Although the comparability of the results was hampered by the large heterogeneity of the study designs and methods, outcome measures, stimulation parameters, and the sites of anodal and cathodal stimulation (21), there also was heterogeneity in cognitive dysfunction in ADHD (61). Based on current evidence, most of the cognitive effects that have been demonstrated are small and insignificant (20, 21). The results of this study can also be interpreted as the learning effect and the repetition effect of tasks. However, tDCS stimulation might also enhance the learning effect. Sham stimuli that were immediately followed by effective stimuli showed better task performance than expected (62). Jacoby and Lavidor (19) also found that the continuous performance task was not affected by tDCS stimulation, and they believed that the learning effect and the repetition effect of the CPT task itself might have an impact on hyperactivity.

The OFC has also been the target region of tDCS research in recent years, although some results have not been particularly promising. For example, some researchers suggested that tDCS reduces resting blood perfusion in the OFC, which is negatively correlated to risky task behavior (63). The tDCS stimulates the OFC, although it has no effect on the impulsivity, exploration of novel things, and risk-taking behaviors of patients with ADHD. However, it may benefit the resistance to new things and avoidance behaviors of OCD patients (64). Recently, a strictly double-blind, randomized, sham-controlled trial was conducted to treat 50 boys with ADHD with right frontal hypothalamus (rIFC) anode tDCS (near the OFC stimulation site) for 15 working days, and combined with cognitive training, the results showed no clinical or cognitive improvement. The findings suggested that rIFC stimulation may not be indicated as a neurotherapy for cognitive or clinical remediation for ADHD (59). However, conclusive evidence from previous tDCS studies in ADHD is mixed by remarkable heterogeneity with respect to stimulus protocol, sample, ADHD symptoms, and cognitive outcome measures. Therefore, this study draws cautious conclusions that, although HD-tDCS does not significantly improve the overall symptoms of patients with ADHD, it can significantly improve their attention maintenance and other neuropsychological deficits. The results further speculated the effect of HD-tDCS in ADHD, indicating that decision-making and impulse control (cognitive and motor control) are complex and interrelated processes and they depend on neural networks containing multiple cortical and subcortical regions, among which the OFC is particularly important. This study also fills the gap in the research of HD-tDCS stimulation of the right OFC.

## Conclusion

This rigorous randomized, sham-controlled trial that had 10 sessions of HD-tDCS was conducted over the right OFC in 47 children and adolescents with ADHD. Although tDCS cannot be recommended as an alternative neurotherapy for ADHD yet, this study draws cautious conclusions that HD-tDCS does not significantly improve the overall symptoms of ADHD patients but leads to significant improvements in cognitive measures of attention maintenance. This study also fills in the research blank of HD-tDCS stimulation of the right OFC.

## Limitations

This study has some limitations, for example, the sample size is relatively small. When patients with ADHD and their parents gave informed consent in the clinic, although experimenters have given full information and explanation, they are still sensitive to words like “electrical stimulation” and even mistake them for “electric shock.” Therefore, they often refused to join the group, which also added to the difficulty of sample collection. A small sample size poses risks that uncontrollable and confounding variables may be unevenly distributed between groups and, thus, potentially affect the results of the experiment. Another example is the higher depigmentation rate. The depigmentation follow-up was analyzed and it was found that, although they only report mild and transient side effects such as tingling or itching of the scalp, mostly not too good stimulation experience is given, which prompted them to depigmentation experiments. This was also reflected in the fact that some of the subjects who completed the experiment showed impatience when the stimulus was administered. Some subjects even reject repetitive and boring cognitive tasks. They think that the whole experiment process of nearly 2 h is tedious, which is also a big factor for the subjects for whom getting medical treatment is difficult. Finally, the interference items were not excluded as much as possible, and the differences in the age of the subjects (age stratification was not achieved) were not distinguished in the grouping. Among the recruited subjects, there were fewer different ADHD subtypes. For example, in this study, there were more subjects with the combined subtype, but fewer subjects with inattention and hyperactive-impulsive subtypes. Therefore, it was not possible to distinguish the differences among the treatment effects of different subtypes. The learning effect and repetition effect were not excluded, and no crossover experimental design was carried out. All of these factors may overestimate or underestimate the effect of the stimulus. The cortical activity of each subject is also different from their own cognitive level, and these confounding factors are also important reasons for the effect of HD-tDCS. Future studies should systematically evaluate the role of interindividual factors (i.e., ADHD subtype, types of the deficit) and stimulation parameters (i.e., site, polarity, intensity, duration, and repetition rate) on tDCS efficacy in the ADHD population (55).

## Data availability statement

The original contributions presented in the study are included in the article/supplementary material, further inquiries can be directed to the corresponding author.

## Ethics statement

The study was approved by the Ethics Committee of Zhenjiang Mental Health Center. Written informed consent to participate in this study was provided by the participants' legal guardian/next of kin.

## Author contributions

Y-cWa and JL conceived, designed this study protocol, and wrote this manuscript. Y-cWa, JL, Y-cWu, YW, H-jX, TZ, and ZZ performed this study. Y-cWa, Y-cWu, and ZZ discussed and conducted data analysis. All authors contributed to data analysis, drafting or revising the article, gave final approval of the version to be published, and agree to be accountable for all aspects of the work.
